# Identification and Biochemical Characterization of a Surfactant-Tolerant Chondroitinase VhChlABC from *Vibrio hyugaensis* LWW-1

**DOI:** 10.3390/md19070399

**Published:** 2021-07-18

**Authors:** Juanjuan Su, Xiaoyi Wang, Chengying Yin, Yujiao Li, Hao Wu, Wengong Yu, Feng Han

**Affiliations:** 1 School of Medicine and Pharmacy, Ocean University of China, 5 Yushan Road, Qingdao 266003, China; sujuanjuan@stu.ouc.edu.cn (J.S.); wangxiaoyi@stu.ouc.edu.cn (X.W.); yinchengying@stu.ouc.edu.cn (C.Y.); lyj6476@stu.ouc.edu.cn (Y.L.); wuhao1330@stu.ouc.edu.cn (H.W.); yuwg66@ouc.edu.cn (W.Y.); 2 Laboratory for Marine Drugs and Bioproducts of Qingdao National Laboratory for Marine Science and Technology, 1 Wenhai Road, Qingdao 266237, China; 3 Key Laboratory of Marine Drugs, Ministry of Education, 5 Yushan Road, Qingdao 266003, China; 4 Shandong Provincial Key Laboratory of Glycoscience and Glycotechnology, 5 Yushan Road, Qingdao 266003, China

**Keywords:** chondroitinases, surfactant tolerance, chondroitin sulfate

## Abstract

Chondroitinases, catalyzing the degradation of chondroitin sulfate (CS) into oligosaccharides, not only play a crucial role in understanding the structure and function of CS, but also have been reported as a potential candidate drug for the treatment of high CS-related diseases. Here, a marine bacterium *Vibrio hyugaensis* LWW-1 was isolated, and its genome was sequenced and annotated. A chondroitinase, VhChlABC, was found to belong to the second subfamily of polysaccharide lyase (PL) family 8. VhChlABC was recombinant expressed and characterized. It could specifically degrade CS-A, CS-B, and CS-C, and reached the maximum activity at pH 7.0 and 40 °C in the presence of 0.25 M NaCl. VhChlABC showed high stability within 8 h under 37 °C and within 2 h under 40 °C. VhChlABC was stable in a wide range of pH (5.0~10.6) at 4 °C. Unlike most chondroitinases, VhChlABC showed high surfactant tolerance, which might provide a good tool for removing extracellular CS proteoglycans (CSPGs) of lung cancer under the stress of pulmonary surfactant. VhChlABC completely degraded CS to disaccharide by the exolytic mode. This research expanded the research and application system of chondroitinases.

## 1. Introduction

Chondroitin sulfate (CS), a kind of glycosaminoglycan that can covalently link with protein to form proteoglycan, is widely distributed in the extracellular matrix and cell surface of animal tissues [1,2]. CS plays an important regulatory role in many physiological processes such as cell development, cell adhesion, proliferation and differentiations [3,4,5,6,7]. Commercially important applications of CS in biological tissue engineering have been explored, which involve combining it with other biopolymers to form scaffolds capable of facilitating and accelerating the regeneration of damaged structures [3].

The CS chain is made up of disaccharide units consisting of d-glucuronic acid (GlcUA)/l-iduronic acid (IdoUA) linked to *N*-acetyl-d-galactosamine (GalNAc) and sulfated at the C2 position of uronic acid and/or at the C4/C6 position of GalNAc residue [8]. Although the structure of the main chain of polysaccharides is not complex, it shows a high degree of heterogeneity in terms of the degree of sulfation, the sulfation position, and the distribution of the two kinds of different isoguronic acids within the chain. Existing studies have indicated the interaction effect between CS and various growth factors/adhesion in numerous important physiological events [8,9,10,11,12]. However, due to the lack of understanding of the fine structure of CS, its functional specificity and interactions with various proteins have been only meagerly explored [5]. Moreover, although CS proteoglycans (CSPGs) are necessary for normal body function, abnormal levels of CSPGs are associated with numerous debilitating conditions [13]. Chondroitinase is mainly used in the pathological condition of increased CSPGs. It can degrade CSPGs to reduce their inhibitory effect on axonal sprouting and functional recovery after spinal cord injury [14]. Another potentially important therapeutic application of chondroitinase is in visual therapy, where it has been shown to induce plasticity in the visual cortex by degrading the deposited CSPGs through animal experiments [15,16]. In addition, the promotion effect of CS on tumor genesis, growth, and metastasis can be neutralized by chondroitinase to a certain extent [17,18]. These all make the chondroitinase indispensable for both structure-function decipherment of CS and deepness development to medicinal value.

Bacteria-derived CS lyases catalyze the degradation of CS via β-elimination reaction, triggering the breaking of 1 → 4 glycosidic bonds between hexosamine and uronic acid residues, and finally forming 4, 5-unsaturated double bonds at the non-reducing end. CS lyases (CSases) can be divided into four classes, CSase ABC, CSase AC, CSase B, and CSase C, according to their substrate specificity. They can also be classified as endo- or exo-type based on the degradation mode [19]. Among these types, the broad catalytic activity of CSase ABCs against CS and hyaluronic acid (HA) determines its crucial applications in both structure–function study and the treatment for many diseases [13,20,21]. However, only a few CSase ABCs derived from bacteria of *Proteus* [19,22], *Bacteroides* [23], *Acinetobacter* [24], and *Sphingomonas* [25] have been identified. The limitation in activity and stability of CSase ABCs have also greatly limited its application. Therefore, it is urgent for both basic research and application to identify more CSase ABCs with better properties.

In this study, a new CSase ABC, named VhChlABC, was identified from the marine bacterium *Vibrio hyugaensis* LWW-1. The main properties and some factors interfering with catalytic reaction of VhChlABC were investigated in detail. With specific degradation for CS, broad tolerance range of pH, and high tolerance to surfactant, VhChlABC might provide a useful tool for CS-related researches and application.

## 2. Results and Discussion

### 2.1. Identification of Strain LWW-1

The 16S rDNA of strain LWW-1 was sequenced and submitted to the 16S-based EzBioCloud’s identification service. Per the results, strain LWW-1 showed the highest similarity of 99.64% with the *Vibrio hyugaensis* strain 090810a (Accession Number: LC004912). Therefore, strain LWW-1 was identified as *V. hyugaensis*.

### 2.2. Identification and Sequence Analysis of CS-Degrading Enzyme

The *vhchlABC* gene (GenBank number: MZ173502) encoding a chondroitin lyase was identified by sequencing the genome of strain LWW-1. It was 3081 bp in length and encoded a 1026-amino-acid protein, including a signal peptide (Met^1^-Asn^24^) at the N-terminus. The theoretical molecular weight of VhChlABC was 114.1 kDa, and the theoretical isoelectric point was 5.59. A conserved GAG lyase domain (Ala^244^-Pro^941^) was predicted in VhChlABC.

**Figure 1:**
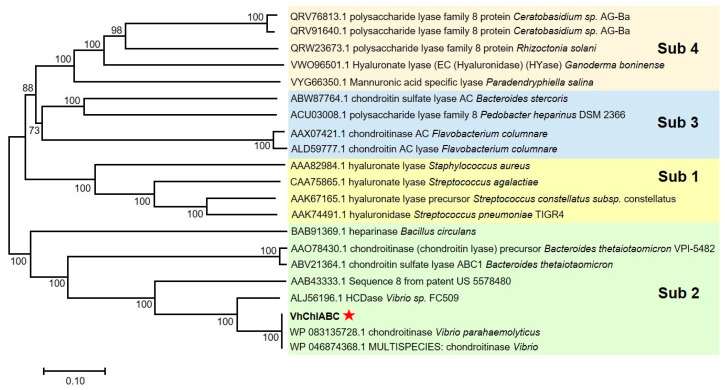
Phylogenetic tree of VhChlABC and other members of PL8. Amino acid sequences were used for this analysis. The numbers (0~100) on the branches indicate the reliability of the corresponding branches. A larger value means more reliable. Sub, subfamily.

**Figure A1:**
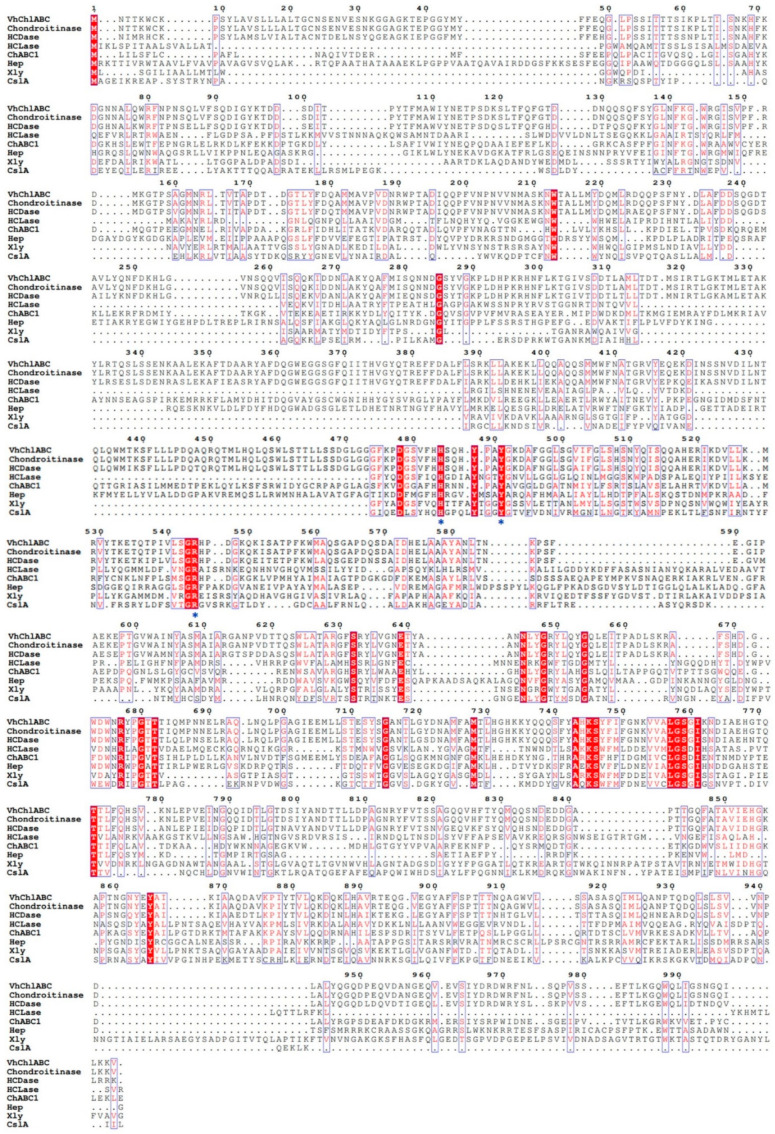
Protein sequence alignment of VhChlABC to the identified enzymes of the PL8 family. Red backgrounds represent the same amino acid residues, and blue frames indicate amino acid residues with identity > 70%. The critical catalytic residues were highlighted by asterisks below them. Chondroitinase, from *Vibrio parahaemolyticus*, GenBank number: WP_083135728; HCDase, from *Vibrio* sp. FC509, GenBank number: ALJ56196.1; HCLase, from *Vibrio* sp. FC509, GenBank number: AIL54323.1; ChABC1, from *Bacteroides thetaiotaomicron* WAL2926, GenBank number: ABV21364.1; Hep, from *Bacillus circulans*, GenBank number: BAB91369.1; Xly, from *Bacillus* sp. GL1, GenBank number: BAB21059.1; CslA, from *Bacteroides stercoris* HJ-15, GenBank number: ABW87764.1.

Similarity analysis indicated that VhChlABC was a member of the PL-8 family, with the highest identity of 82.05% to the characterized HCDase (GenBank number: ALJ56196.1) from *Vibrio* sp. FC509 [26]. A phylogenetic tree was drawn based on the amino acid sequences of VhChlABC and other characterized members of the PL-8 family. As a result, VhChlABC was assigned to the second subfamily (Figure 1). The amino acid alignment of VhChlABC and the identified PL8 family enzymes showed that VhChlABC contained the conserved catalytic residues of the PL8 family in His^484^, Tyr^491^, and Arg^544^ (Figure A1 in Appendix B).

### 2.3. Recombinant Expression and Purification of VhChlABC

**Figure 2:**
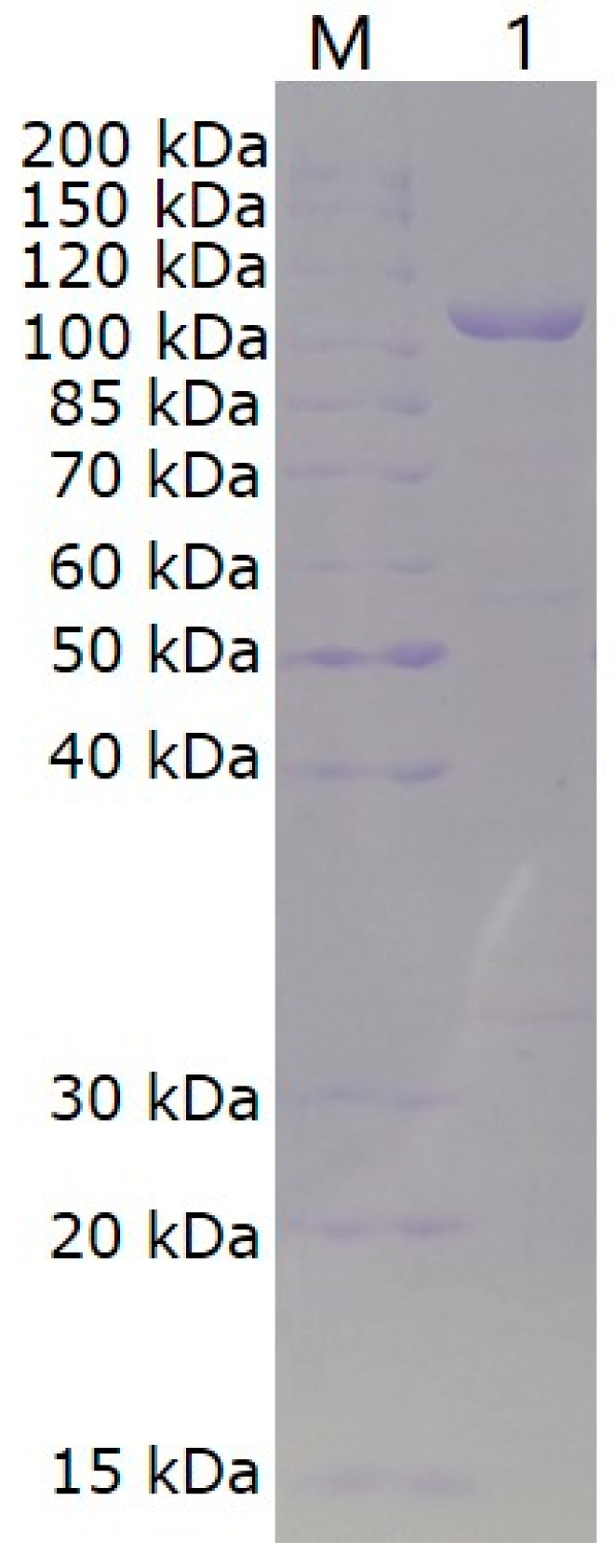
SDS-PAGE of VhChlABC. Lane M, protein marker; lane 1, purified VhChlABC.

The gene *vhchlABC* was cloned into the vector pET-28a (+) with removal of the signal peptide and addition of (His)_6_-Tag at both ends. VhChlABC was expressed in soluble forms in the pET-28a (+)/*E.coli* BL21 (DE3) system. The recombinant VhChlABC was obtained from 40 mL of the bacteria lysate supernatant through nickel affinity chromatography. As shown in Figure 2, a main band (estimated purity > 90%) in SDS-PAGE with a molecular weight of about 110 kDa was identified. The specific activity of purified VhChlABC towards CS-A was 17.54 U/mg. The protein recovery was 16.69% during purification, and about 9.25 mg of purified VhChlABC could be obtained from 1 L of bacterial fermentation broth (Table 1).

### 2.4. Substrate Specificity of VhChlABC

**Figure 3:**
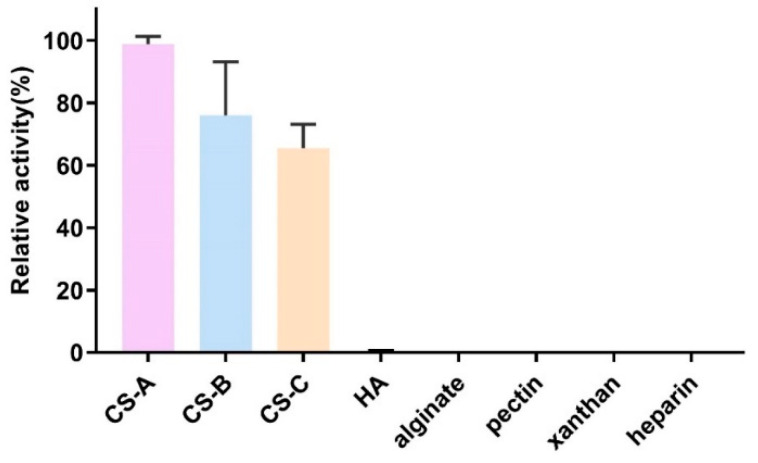
Substrate specificity of VhChlABC. The activity of VhChlABC towards CS-A was defined as 100%. Error bars indicate standard deviation (*n* = 3).

The substrate specificity of VhChlABC was determined using a variety of glycolaminoglycans as substrates. As shown in Figure 3, VhChlABC could only degrade CS-A, CS-B, and CS-C, but showed no activity towards other tested substrates. VhChlABC showed the highest degradation activity towards CS-A, and the lowest towards CS-C, which was more than 60% of the former. The specific activity towards CS-A and CS-B was similar. Among the three kinds of CS, VhChlABC possessed the highest *k_cat_*/*K_m_* value on CS-A at 1.83 min^−1^·mM^−1^, followed by CS-C and CS-B (Table 2).

Most reported bacteria-derived CSase ABC exhibit a wide range of degrading activity against HA and CS, such as HCDase and HCLase from *Vibrio* sp. FC509 [26,27], chondroitin sulfate ABC lyase (EC 4.2.2.20) from *Bacteroides thetaiotaomicron* WAL2926 [23], ChSase ABC from *Acinetobacter* sp. C26 [24], ChSase ABC from *Sphingomonas paucimobilis* [25], and chondroitinase ABC I and chondroitinase ABC II (EC 4.2.2.20) from *Proteus vulgaris* et al. [19,22]. Unlike these, VhChlABC could degrade CS but not HA, which was also found in the study of ChABC I and ChABC II from *Edwardsiella tarda* LMG2793 and of chondroitin ABC from *Bacteroides stercoris* [28,29].

### 2.5. Effects of Temperature and pH on VhChlABC

**Figure 4:**
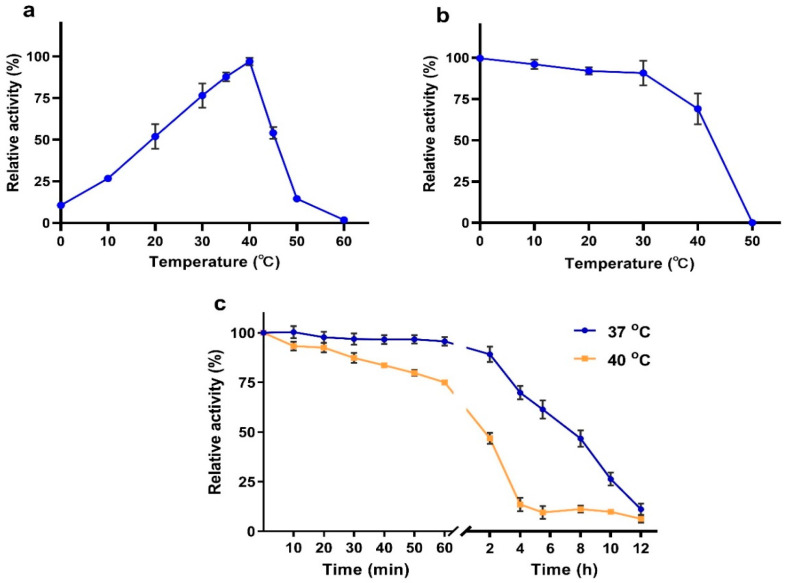
Effects of temperature on VhChlABC. (**a**) Optimal temperature of VhChlABC. (**b**,**c**) The thermostability of VhChlABC. (**b**) The residual activity of VhChlABC was measured after pre-incubation at different temperatures (0~50 °C) for 1 h. (**c**) The residual activity of VhChlABC was detected at different timepoints after incubation at 37 °C and 40 °C. The initial activity was defined as 100%. Error bars indicated standard deviation (*n* = 3).

According to the results of an optimal temperature test, VhChlABC exhibited the highest activity towards CS-A at 40 °C (Figure 4a), which was similar to most members of PL-8 family, such as ChSase ABC from *Bacteroides stercoris* and *Sphingomonas paucimobilis* or ChSase AC from *Flavobacterium heparinum* et al. [25,29,30]. Thermostability testing indicated that after pre-incubation at a temperature below 30 °C for 1 h, VhChlABC remained about 90% of its initial activity (Figure 4b). After pre-incubation at 40 °C for 1 h (Figure 4b), the activity retained was more than 60%, and it was approximately 50% after pre-incubation for 2 h (Figure 4c). However, the activity decreased rapidly when the pre-incubation temperature was higher than 40 °C (Figure 4b). Furthermore, after incubation for more than 2 h at 40 °C, the activity of VhChlABC decreased rapidly and was almost lost until 6 h (Figure 4c). After treatment at 37 °C for 8 h, VhChlABC could retain about 50% of its initial activity; however, the activity reduced rapidly and was almost lost after 12 h (Figure 4c). In fact, poor thermostability has become an important limiting factor for the application of CS lyases. As reported, cABC I and cABC II from *Proteus vulgaris* NCTC 4636 cannot maintain 50% of their activity after treatment at 40 °C for 30 min [31]. As for HCDase from *Vibrio* sp. FC509, its activity gradually declined to 20% after treatment at 30 °C for 4 h [26]. In this research, VhChlABC presented higher stability within 8 h under 37 °C (physiological temperature) and within 2 h under 40 °C (optimal temperature), which might contribute to CS-related clinical application and industrial production.

**Figure 5:**
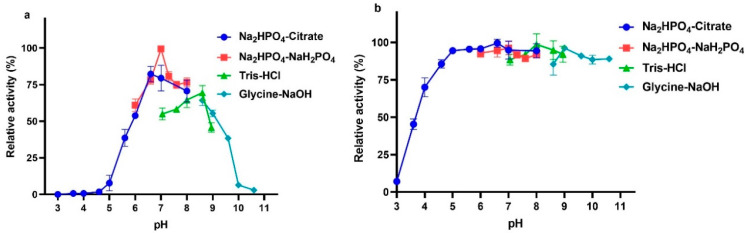
Effects of pH on VhChlABC. (**a**) Optimal pH of VhChlABC. (**b**) pH stability of VhChlABC. CS-A was used as the substrate. The activity of VhChlABC at the optimal pH and temperature was defined as 100%. Error bars indicated standard deviation (*n* = 3).

The optimal pH value for the degradation activity of VhChlABC against CS-A was pH 7.0 in 50 mM Na_2_HPO_4_-NaH_2_PO_4_ buffer (Figure 5a). Moreover, VhChlABC could keep stable in different buffers of pH 5.0~10.6 (Figure 5b). With the high stability on pH, VhChlABC might be easier to preserve and more widely used in the industry.

### 2.6. Effects of Metal Ions, Chelators, and Surfactants on VhChlABC

**Figure 6:**
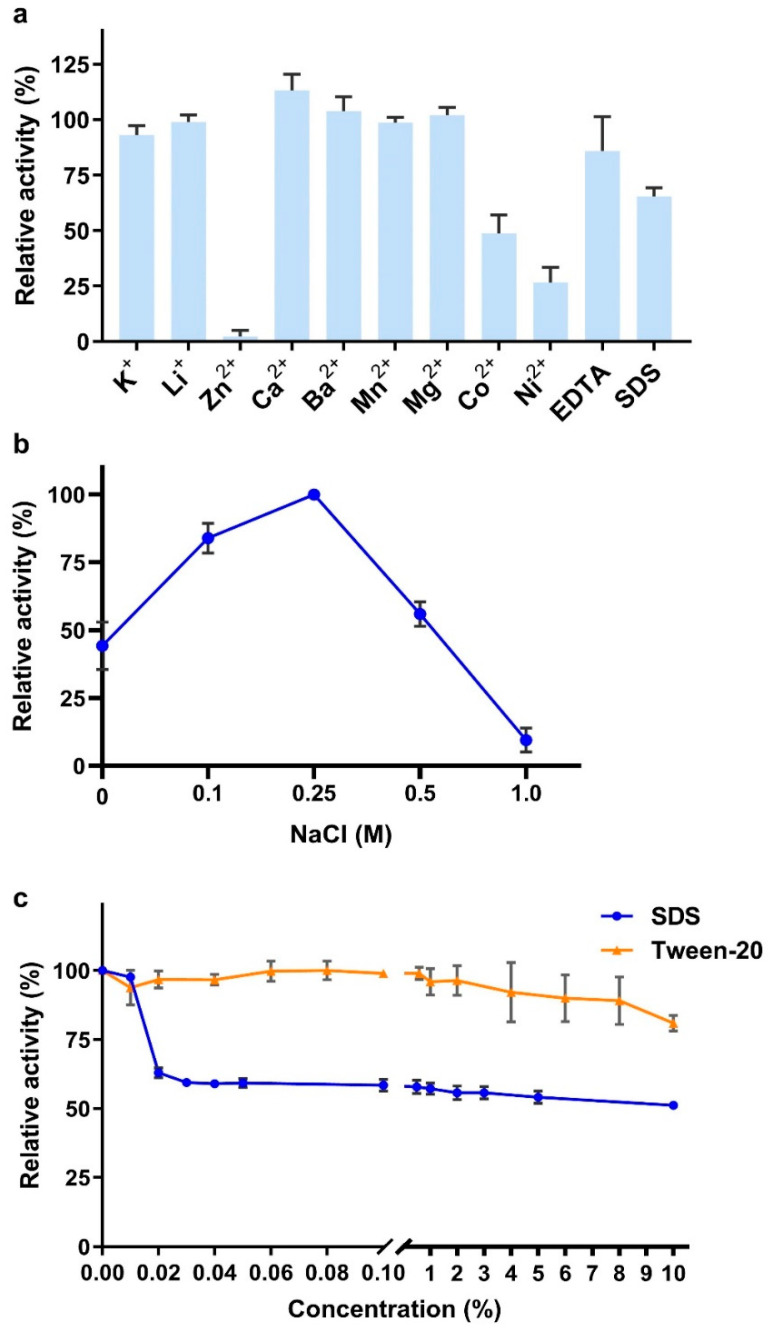
Effects of metal ions, chelators, and detergents on the activity of VhChlABC. (**a**) Effects of metal ions, chelator (1 mM), and surfactant (SDS, 0.1%, *w*/*v*). (**b**) Effects of NaCl concentrations (0~1 M). (**c**) Effects of SDS and Tween-20. Error bars indicated standard deviation (*n* = 3).

As shown in Figure 6a, no tested metal ions enhanced the CS degradation activity of VhChlABC significantly. Zn^2+^, Co^2+^, and Ni^2+^ could strongly inhibit its activity, which was consistent with *Vibrio* sp. FC509 HCDase and HCLase-related results [26,27]. The structural changes caused by the affinity of heavy metals towards the SH, CO, and NH groups in the amino acids might be the crucial factor for the inhibition of VhChlABC by metal ions such as Zn^2+^ [32]. In addition, differences of amino acids on the protein surface might also be important for the metal ion effect [33], which might result in the different effects of metal ions on HCDase, HCLase, and VhChlABC. Additionally, NaCl enhanced the CS-degradation activity of VhChlABC at low concentrations (≤0.25 M) and exhibited the maximum effect at 0.25 M (Figure 6b). Notably, even if there was no NaCl in the reaction system, VhChlABC still maintained nearly 50% of its maximum activity (Figure 6b), which indicated that VhChlABC had no dependency on NaCl and could satisfy more NaCl-limited production conditions.

In particular, VhChlABC well tolerated both SDS and Tween-20, maintaining more than 50% of its maximum activity in the presence of up to 10% (*w*/*v*) SDS and having almost no loss of activity in the presence of up to 10% (*v*/*v*) Tween-20 (Figure 6c). Although research has continued to report glycosaminoglycanases (GAGases) of novel structures and functions, studies on surfactant-tolerant GAGases are almost nonexistent. Besides being used as detergents in daily life, other applications of surfactants could cover almost all fine chemical fields [34]. Unfortunately, most of the reported GAGases were inactivated in the presence of very low concentrations of SDS. As reported, SDS could almost completely inhibit the activity of enCSase from *Photobacterium* sp. QA16 [20], Vpa_0049 from *Vibrio* sp. QY108 [35], and ChSase ABC from *Acinetobacter* sp. C26 [24], with final concentrations of 5 mM, 1 mM, and 5 mM, respectively. Unfortunately, the surfactant tolerance of many enzymes has not been measured, including HCDase from *Vibrio* sp. FC509. The crucial domains and key amino acid residues that determine the surfactant tolerance of VhChlABC must be further explored.

It was considered a safe and effective strategy to remove extracellular CS proteoglycans (CSPGs) using chondroitinase, which was reported to promote viral spread and infection in oncolytic virus (OV)-mediated treatment of astrocytomas [36]. However, the sensitivity of chondroitinase to trace surfactants might limit its application in the lung environment under the stress of pulmonary surfactant. Therefore, the high surfactant tolerance of VhChlABC might open a new horizon for the treatment of lung cancer. In addition, *Vibrio* is widely distributed in different water bodies and marine animals, has strong vitality, and has been recognized as an important pathogen in the aquaculture industry [37]. The identification of the surfactant-tolerance gene *vhchlABC* is of great significance for understanding the stress tolerance mechanism of *Vibrio*.

### 2.7. Degradation Mode and End Products of VhChlABC

**Figure 7:**
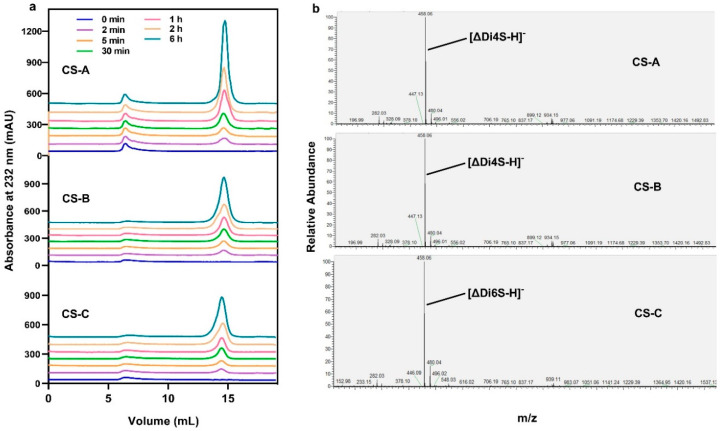
The reaction modes and end products of CS-A, CS-B, and CS-C degraded by VhChlABC. (**a**) The time courses of CS-A, CS-B, and CS-C degradation by VhChlABC. (**b**) ESI-MS analysis of the end products.

SEC was performed to analyze the degradation modes and end products of CS-A, CS-B, and CS-C by VhChlABC. Per the results, only one major product peak was detected during the reaction (Figure 7a), indicating that VhChlABC degraded CS through the exolytic mode, which was consistent with HCDase [26]. All the enzymatic properties suggested that VhChlABC was an EC 4.4.2.21—chondroitin-sulfate-ABC exolyase. The end products of CS-A, CS-B, and CS-C were all identified as the same single peak (data not shown), with a molecular weight of 458.06 *m/z* (*z* = 1) identified by negative-ion ESI-MS (Figure 7b). It was consistent with the m/z of unsaturated monosulfated disaccharides of CS-A ([ΔDi4S-H]^−^), CS-B ([ΔDi4S-H]^−^), and CS-C ([ΔDi6S-H]^−^) [25,35]. These results indicated that VhChlABC could completely degrade CS to unsaturated disaccharides through the exolytic mode.

## 3. Materials and Methods

### 3.1. Reagents

Phanta max super-fidelity DNA polymerase (P505-d1) was purchased from Vazyme (Nanjing, China). Takara quick-cut enzyme and T4 DNA ligase kit were purchased from Biomedical Technology Co., Ltd. (Beijing, China). Chondroitin sulfate A (CS-A), CS-B, and CS-C were purchased from Hefei Bomei Biotechnology Co., Ltd. (Hefei, China). HA, alginate, pectin, xanthan, and heparin were purchased from Solarbio (Beijing, China). TIANamp bacteria DNA kit (Tiangen, China) was used to extract the bacterial genome. HisTrap HP column and Superdex Peptide 10/300 GL was purchased from GE Healthcare (Pittsburgh, PA, USA).

### 3.2. Isolation of Marine CS Lyase-Producing Bacteria

Seawater sample was collected from Zhanqiao, QingDao, China. A selective medium, which consisted of 0.3% (*w*/*v*) KH_2_PO_4_, 0.7% (*w*/*v*) K_2_HPO_4_·3H_2_O, 0.2% (*w*/*v*) (NH_4_)_2_SO_4_, 0.01% (*w*/*v*) FeSO_4_·7H_2_O, 3% NaCl, 0.05% (*w*/*v*) CS (CS-A: CS-B: CS-C = 1:1:1 by mass), and 1.5% (*w*/*v*) agar (pH 7.0), was used to isolate CS lyase-producing bacteria from the seawater sample. Obviously, CS was the sole carbon source. The screening model referred to the Gram’s iodine plate assay method for hyaluronidase [38]. Briefly, after pre-incubation at 25 °C for 48 h, the plates containing the microorganisms were soaked with Gram’s iodine for 1 min. Clones with distinct clearance zones were detected as CS-degrading strains and then purified with fresh selective medium plates three times to obtain the pure cultured strains. Then, the pure monoclonal strains were cultured in 100 mL marine broth 2216 at 25 °C and 160 rpm/min for 48 h, and the CS lyase activity in the culture supernatant was determined using the A_232_ method (as shown in Section 3.6). The strain LWW-1, which exhibited the highest CS lyase activity, was obtained and used in the following experiment.

### 3.3. Identification of Strain LWW-1

The 16S rDNA of strain LWW-1 was amplified by PCR with the universal primer pairs 27F (5′-AGAGTTTGATCCTGGCTCAG-3′)—1492R (5′-TACGGTTACCTTGTTACGACTT-3′), using a colony strain as the template. After purification, the PCR product was then sequenced by Ruibiotech Co., Ltd. (Beijing, China). The isolated strain LWW-1 was identified using 16S rDNA sequence using the EzBioCloud’s identification service (https://www.ezbiocloud.net accessed on 15 November 2020).

### 3.4. Sequence Analysis of VhChlABC

The genomic DNA of strain LWW-1 was prepared using Tianamp bacteria DNA kit. The genome of strain LWW-1 was sequenced by Novogene Bioinformatics Technology Co., Ltd. (Beijing, China) and then annotated with Rapid Annotation using Subsystem Technology (RAST). Sequence similarity analysis was performed by online BLAST. The phylogenetic tree was drawn using the neighbor-joining method by MEGA 7.1. The molecular weight (Mw) and isoelectric point (pI) were analyzed by the compute Mw/pI tool on ExPASy of Swiss Bioinformatics Resource Portal (http://us.expasy.org/tools/pi_tool.html accessed on 15 November 2020). The SignalP 5.0 server (http://www.cbs.dtu.dk/services/SignalP/ accessed on 15 November 2020) was used to predict the signal peptide and its cleavage site in VhChlABC. The protein domains within VhChlABC were identified using the Conserved Domains server of NCBI database (http://www.ncbi.nlm.nih.gov/Structure/cdd/wrpsb.cgi accessed on 15 November 2020). Amino acid alignment with other enzymes of the PL8 family was carried out using ESPrit 3.0 (https://espript.ibcp.fr/ESPript/ESPript/ accessed on 15 November 2020).

### 3.5. Recombinant Expression and Purification of VhChlABC

The full-length gene of *vhchlABC* without the signal peptide sequence was amplified with super-fidelity DNA polymerase (Vazyme, Nanjing, China) and the primer pairs ChlABC-F (GTACcatatgAGCGAAAATGTTGAAAGCA) and ChlABC-R (GTACctcgagTACTTTTTTCAGCATGAATTTT), taking the genomic DNA of *V. hyugaensis* LWW-1 as the template. After digestion with *Nde* I and *Xho* I, the gel-recovered PCR products were ligated into plasmid pET-28a (+). Then, the plasmid pET-28a-VhChlABC was transformed into *E. coli* BL21 (DE3) cells to obtain the recombinant expression strains.

The recombinant expression strains were cultured in Luria–Bertani (LB) medium (containing 30 μg/mL kanamycin) with shaking at 37 °C until the OD_600_ value reached 0.4~0.6. Then, it was induced to express VhChlABC by adding 0.1 mM isopropyl-1-thio-β-d-galactoside (IPTG) and incubated at 18 °C for 24 h. After centrifugation with 8000 rpm/min for 30 min at 4 °C, cells were resuspended in precooled 20 mM phosphate buffer (PB) containing 500 mM NaCl at pH 7.0 (binding buffer) at a volume of 1/10 of the initial fermentation broth, then ruptured by the high-pressure cell cracker at 4 °C. Next, the supernatant (usually named crude extract) containing the soluble target protein was collected by centrifugation at 12,000 rpm/min for 30 min. The crude extract was subsequently filled by a constant current pump onto a Ni-Sepharose column (GE Healthcare, Pittsburgh, PA, USA) pre-balanced with binding buffer. The (His)_6_-tagged VhChlABC protein was eluted from the column using binding buffer with 300 mM imidazole added. The molecular weight and purity of VhChlABC were examined by SDS-PAGE combined with Coomassie Bright Blue staining. Protein content was measured according to the instructions of BCA protein assay kit (EpiZyme, Shanghai, China).

### 3.6. Enzyme Activity Determination of VhChlABC

In this study, two methods were used to determine the enzyme activity. As for the A_232_ method [28], a reaction system containing 100 μL of enzyme sample, which in turn contained approximately 1.0 μg of purified VhChlABC, and 900 μL of 0.2% (*w*/*v*) CS substrate was incubated at 40 °C for 10 min. The change of absorbance value at 232 nm was measured to quantify the unsaturated double bonds in the system. One unit (U) was defined as the amount of enzyme required to catalyze the production of 1 μmol of 4,5-unsaturated uronic acid per minute. The millimolar absorption coefficients for CS-A, CS-B, and CS-C were 5.1, 5.1, and 5.5 respectively [39].

As for the DNS (dinitrosalicylic acid) method [40], 50 μL of enzyme sample, which contained approximately 3.0 μg of purified VhChlABC, and 450 μL of 0.2% (*w*/*v*) CS substrate (20 mM PB, pH 7.0) were co-incubated at 40 °C for 10 min. After that, 375 μL of DNS reagent, prepared as described in Appendix A, was added to the system and boiled at 100 °C for 10 min. Then, the reducing sugar content was quantified by measuring the absorbance value of the reaction solution at 520 nm using d-glucosamine as a standard. For both methods, the same reaction with an equal amount of enzyme, inactivated by boiling for 10 min, was used as the blank. One unit was defined as the amount of enzyme that generated reducing sugars corresponding to 1 μmol of glucosaminehydrochloride per minute under standard conditions.

### 3.7. Substrates Specificity Analysis of VhChlABC

Various commercial polysaccharide substrates at 0.2% (*w*/*v*), including HA, CS-A, CS-B, CS-C, alginate, pectin, xanthan and heparin (20 mM PB, pH 7.0), were used as substrates to determine the substrate specificity of VhChlABC. A_232_ method was used for this test under optimal conditions.

### 3.8. Biochemical Characterization of VhChlABC

Biochemical characterization was determined using CS-A as the substrate. The A_232_ method was used for this test. For optimum temperature detection, 0.2% (*w*/*v*) CS-A (20 mM PB, pH 7.0) was digested by VhChlABC at a serial temperature gradient from 0 to 60 °C. To determine the thermostability, VhChlABC was pre-incubated at different temperatures (0~50 °C) for 1 h, and the residual activity was measured at 40 °C. Furthermore, the residual activity of VhChlABC was examined at different times post incubation at 37 °C and 40 °C. The initial specific activity detected at 40 °C was set as 100%.

For optimum pH detection, the activity of VhChlABC was analyzed under different pH buffers of 50 mM at 40 °C, including Na_2_HPO_4_-NaH_2_PO_4_ with pH of 6.0~8.0, Tris-HCl with pH of 7.05~8.95, NaH_2_PO4-citric acid with pH of 3.0~8.0, and glycine-NaOH with pH of 8.6~10.6. To analyze the effect of pH on stability, VhChlABC was incubated in a series of pH buffers at 4 °C for 12 h, respectively. Then, residual activity was measured by the A_232_ method under optimal conditions.

Different ions, including K^+^, Li^+^, Zn^2+^, Ca^2+^, Ba^2+^, Mn^2+^, Mg^2+^, Co^2+^, and Ni^2+^, and chelator (EDTA) were separately added to the reaction mixture at a final concentration of 1 mM to detect their effect on CS degradation by VhChlABC. NaCl dependence might be the specificity of marine active enzymes; therefore, the effects of NaCl concentrations (0~1.0 M) on the activity of VhChlABC were detected in this research. The A_232_ method was used for the above detection under optimal conditions. The effects of SDS and Tween-20 on VhChlABC were detected by adding different final concentrations of SDS (0~10%, *w*/*v*) and Tween-20 (0~10%, *v*/*v*) separately into the reaction system. SDS and Tween-20 can greatly increase the absorbance of the substrate at 232 nm, which is beyond the reliable range of the A_232_ method. Therefore, the DNS method was used for this detection.

### 3.9. Reaction Kinetics of VhChlABC towards CS

CS-A, CS-B, and CS-C were each dissolved in 20 mM PB (pH 7.0) to prepare substrates with concentrations from 0.2 to 2.0 mg/mL. The absorbance value of the enzyme-substrate (1:9 by vol) reaction system at 232 nm was measured after incubation at 40 °C for 3 min. *V_max_* and *K_m_* values were calculated using the Michaelis–Menten equation and the curve fitting program by the non-linear regression analysis using Graphpad Prism 8. The *k_cat_* value was the ratio of *V_max_* to the enzyme concentration.

### 3.10. Reaction Mode and End Products of VhChlABC

The reaction modes of VhChlABC towards CS-A, CS-B, and CS-C were monitored. Degradation reaction was initiated by uniformly mixing enzyme and substrate (20 mM PB, pH 7.0) in proper proportions under optimal conditions and terminated by boiling at different timepoints. The unsaturated oligosaccharide products with different degrees of polymerization in the samples of different timepoints were separated and analyzed by size-exclusion chromatography (SEC). Fast protein liquid chromatography (FPLC, GE Healthcare, Pittsburgh, PA, USA) combined with a Superdex peptide 10/300 GL column (GE Healthcare, Pittsburgh, PA, USA) was used for this process. For the mobile phase, 0.2 M NH_4_HCO_3_ at a flow rate of 0.2 mL/min was used. The absorbance value at 232 nm was monitored.

Moreover, 0.5 mL CS-A/CS-B/CS-C (0.2%, *w*/*v*, 20 mM PB, pH 7.0) was co-incubated with 0.5 mL purified VhChlABC (containing approximately 20 μg of purified VhChlABC) at 40 °C for 24 h. The end-products were detected and separated by SEC, then further identified by negative-ion electrospray ionization-mass spectrometry (ESI-MS).

## 4. Conclusions

In this study, a novel PL-8 chondroitin sulfate lyase, VhChlABC, was identified and characterized from marine bacterium *V. hyugaensis* LWW-1. Although VhChlABC has the highest amino acid identify (82.05%) with HCDase, it possesses some significantly different characteristics from the latter. The discovery of surfactant-tolerant chondroitinase provides a new way for the treatment of CS-related diseases in special pathological conditions. The wider pH tolerance range makes VhChlABC a potentially good enzymatic tool in industry application without rigorous reaction and storage conditions.

## Figures and Tables

**Table 1:** Summary of the purified VhChlABC.

Step	Specific Activity (U/mg)	Total Protein (mg)	Total Activity (U)	Fold Purification	Yield (%)
Fermentation broth	3.66	106.22	388.77	1	100
Nickel column	17.54	3.70	64.90	4.79	16.69

The A_232_ method was used for this detection. Enzyme activity was measured using 0.2% CS-A as substrate (20 mM PB, pH 7.0) under optimal conditions. The volume of the initial fermentation broth was 400 mL, and the volume of the concentrated crude extract was 40 mL.

**Table 2:** *K_m_*, *k_cat_*, and *V_max_* values of VhChlABC.

Substrate	*K_m_* (μM)	*V_max_* (μmol·min^−1^)	*k_cat_* (min^−1^)	*k_cat_*/*K_m_* (min^−1^·mM^−1^)
CS-A	2.90 ± 0.35	1.06 ± 0.04	5310 ± 200	1.83 ± 0.069
CS-B	4.29 ± 0.33	0.90 ± 0.02	4468 ± 123	1.04 ± 0.029
CS-C	2.67 ± 0.22	0.70 ± 0.01	3465 ± 72	1.30 ± 0.027

## Appendix A

Preparation method of DNS reagent. 1. Preparation of solutions A and B. Solution A: 6.9 g crystalline phenol was dissolved in 15.2 mL of 10% NaOH solution and diluted to 69 mL with water, and then 6.9 g NaHSO_3_ was added. Solution B: 255 g NaKC_4_H_4_O_6_·4H_2_O was dissolved in 200 mL of 10% NaOH solution, and then 880 mL of 1% DNS solution was added. 2. Mix solutions A and B and allow the reagent to stand for 7 to 10 days at room temperature before use.
